# Editorial: Membrane Trafficking in Immunology - How Membrane Transport and Exocytosis Defects Underlie Immunodeficiencies

**DOI:** 10.3389/fimmu.2021.769815

**Published:** 2021-09-30

**Authors:** Paul T. Manna, Samuel C. C. Chiang, Yenan T. Bryceson, Jordan S. Orange, Sandra Ammann

**Affiliations:** ^1^ Institute of Neuroscience and Physiology, University of Gothenburg, Gothenburg, Sweden; ^2^ Division of Bone Marrow Transplantation and Immune Deficiency, Cincinnati Children’s Hospital Medical Center, Cincinnati, OH, United States; ^3^ Department of Pediatrics, University of Cincinnati, Cincinnati, OH, United States; ^4^ Center for Hematology and Regenerative Medicine, Department of Medicine, Karolinska Institutet, Stockholm, Sweden; ^5^ Primary Immunodeficiency Unit, Clinical Immunology and Transfusion Medicine Clinic, Karolinska University Hospital Huddinge, Stockholm, Sweden; ^6^ Broegelmann Research Laboratory, Department of Clinical Science, University of Bergen, Bergen, Norway; ^7^ Department of Pediatrics, Vagelos College of Physicians and Surgeons, Columbia University Irving Medical Center, New York, NY, United States; ^8^ Institute for Immunodeficiency, Center for Chronic Immunodeficiency (CCI), Medical Center - University of Freiburg, Faculty of Medicine, University of Freiburg, Freiburg, Germany

**Keywords:** membrane trafficking, primary immunodeficiencies (PID), inborn errors of immunity, STING, cytotoxic T cells

The membrane trafficking system establishes and maintains the correct distribution and compartmentalisation of proteins within the cell. Broadly speaking, membrane trafficking processes encompass the selection of protein cargoes, their packaging into transport intermediates, and the delivery and fusion of these transport intermediates with the proper acceptor organelle. As such, this machinery functions constantly and ubiquitously within almost every cell of the body. Perhaps counterintuitively then, given the ubiquity of these processes, a large and increasing number of pathogenic mutations affecting the membrane trafficking machinery have been identified with relatively restricted pathologies (1). Notable among these are a range of immunological disorders.

Often considered at the level of cellular interactions and their associated receptors and signalling pathways, both the innate and adaptive arms of the immune system rely upon subcellular membrane trafficking events to support many essential functions. These include the biosynthesis and proper subcellular targeting of immune receptors, phagocytic uptake and killing of invading pathogens, formation and targeted delivery of specialised lytic organelles in T and NK cells, and modulation of signalling *via* dynamic translocation of receptors and signalling partners. As such, genetic mutations affecting proteins of the membrane trafficking machinery underlie diverse disorders with immunological components, including familial haemophagocytic lymphohistiocytosis (MUNC-13-4, SYNTAXIN 11, MUNC18-2, RAB27A, RHOG) (2), systemic lupus erythematosus (LTK) (3), Chediak-Higashi syndrome (LYST), and forms of Hermansky-Pudlak syndrome (AP3, BLOC complex components) (4).

The articles within this special Research Topic focus on just two aspects of membrane trafficking in immunity, namely the formation and release of lytic granules from cytotoxic cells and the regulation of DNA-sensing pattern recognition receptors through their proper subcellular targeting. These articles investigate the genetic and mechanistic basis of diseases associated with these processes and further explore the pathophysiological implications, both in terms of the primary immunological defects and in a particularly detailed review by Mastio and Saeed et al. the implications for the development of downstream malignancies.

## Exocytosis of Lytic Granules Leading to Inborn Error of Immunity

Impaired cytotoxic function due to reduced secretory granule exocytosis by NK and CD8^+^ T cells results in uncontrolled hyperactivation of macrophages and T cells. Clinically, this manifests as familial hemophagocytic lymphohistiocytosis (fHLH) (5). Numerous autosomal recessive mutations affecting granule exocytosis have been identified (*UNC13D, STXPB2, STX11, LYST, RAB27A, RHOG, AP3B1, AP3D1*). Characterisation of these mutants has given great insight into secretory granule transport, docking, priming and fusion (reviewed in Mastio and Saeed et al). However, the potential role of heterozygous mutations is still debated. ***N.*** Benavides and A. Spessott et al. investigate the effects of a heterozygous mutation (R190C) in *syntaxin-binding protein-2* (*STXBP2)* identified accompanied by a hemizygous *glucose 6-phosphate dehydrogenase* (*G6PD*, C385R) mutation. They show that the heterozygous mutation does not interfere with protein or mRNA stability, nor interaction with syntaxin-11 (STX11). Since this protein region is conserved, they hypothesize binding to an unknown interaction partner. Overexpression in healthy control CD8^+^ T cells reduced secretory granule exocytosis dramatically, suggesting a dominant-negative effect of this mutation. This supports the growing evidence that heterozygous mutations in synergy with other mutations could lead to fHLH manifestation. Importantly, family members carrying the heterozygous R190C mutation in *STXPB2* should be excluded as hematopoietic stem cell donors.

Most homozygous mutation of RAB27A described to date predispose to HLH with albinism. Ohishi et al investigate an undescribed homozygous mutation, *RAB27A* p.(*Val143Ala)*, identified in a patient with HLH without any associated albinism. They demonstrate that RAB27A *Val143Ala* does not affect the RAB27A–melanophilin (MLPH)/SLAC2-A interaction, essential for hair and skin pigmentation. However, in line with some previously characterised RAB27A mutations, CD8^+^ T cell function is impaired and reduced binding activity towards MUNC13-4 and SLP2-A is suggested. The authors summarize other *RAB27A* mutations *sine* albinism to demonstrate the importance of *RAB27A* sequencing in patients with suspected HLH without pigmentary dilution.

Calcium plays a regulatory role in serial secretory granule exocytosis and killing function of CD8^+^ T cells. Sleiman et al. suggest a role for Synaptotagmin7 (SYT7), a high affinity calcium sensor, in the transport of secretory granules to the immunological synapse (IS), especially during the second phase of prolonged secretory granule fusion. They demonstrate that *SYT7* KO CD8^+^ T cells display a conditional granule fusion defect under high 10mM [Ca^2+^] conditions, rescued by transduction of *SYT7* wild type constructs.

## IEI Affecting Membrane Trafficking and Promoting Cancer Development

Whilst the primary immunological defect is often the best understood in terms of mechanism and pathogenesis it is important to remember that downstream sequalae may contribute significantly to a patient’s clinical needs. Mastio and Saeed et al. provide a review highlighting the links between membrane trafficking defects, their consequent immunological deficiencies, and the subsequent development of B-cell malignancies. They detail the processes leading to malignancies and explore the involvement of actin regulators and immune synapse defects in transformation and tumor surveillance.

## Regulation of DNA-Sensing Pattern Recognition Receptors *via* Dynamic Translocation

A focus of much attention in recent years has been the role of subcellular compartmentalisation in the innate immune response to so-called pathogen associated molecular patterns (PAMPs). Two reviews in the current research focus examine the critical regulation of DNA-sensing pattern recognition receptors *via* their dynamic translocation between subcellar compartments. Taguchi and Mukai et al. give an overview of current knowledge concerning STING translocation between the ER and Golgi and highlight a recently described autoinflammatory disorder (COPA syndrome) arising from mutations in the α-COP subunit of the COP-I coat. In a complementary review Amadio et al. build a case for more detailed exploration of the role of the actin machinery in regulating STING and TLR9 distribution and function. They discuss recent evidence of aberrant DNA sensing associated with the actin-related primary immuno-deficiency Wiskott-Aldrich syndrome and draw links to the roles of actin both upstream, and downstream of DNA sensing by STING and TLR9.

## End Note

Together, the articles contained in this special Research Topic give insight into two of the many important roles that membrane trafficking plays in maintaining the proper function of the immune system. Further exploration of this subject will be greatly aided by continuing advances in both the medical genetics of immunodeficiency as well as experimental approaches to the study of membrane trafficking and the manipulation of immune cells. As such we believe that this will be a growing and important field of investigation in the coming years.

## Author Contributions

All authors listed have made a substantial, direct, and intellectual contribution to the work and approved it for publication.

## Funding

Funding SFB 1160/IMPATH for SA and YTB is supported by the Swedish Research Council, Cancer Foundation, Childhood Cancer Foundation, the Knut and Alice Wallenberg Foundation, the Center for Innovative Medicine and Stockholm Region.

## Conflict of Interest

The authors declare that the research was conducted in the absence of any commercial or financial relationships that could be construed as a potential conflict of interest.

## Publisher’s Note

All claims expressed in this article are solely those of the authors and do not necessarily represent those of their affiliated organizations, or those of the publisher, the editors and the reviewers. Any product that may be evaluated in this article, or claim that may be made by its manufacturer, is not guaranteed or endorsed by the publisher.
